# How stands the Tree of Life a century and a half after *The Origin*?

**DOI:** 10.1186/1745-6150-6-32

**Published:** 2011-06-30

**Authors:** Maureen A O'Malley, Eugene V Koonin

**Affiliations:** 1 Department of Philosophy, Quadrangle A14, University of Sydney, NSW 2006, Australia; 2 National Center for Biotechnology Information, National Library of Medicine, National Institutes of Health, Bethesda MD20894, USA

## Abstract

We examine the Tree of Life (TOL) as an evolutionary hypothesis and a heuristic. The original TOL hypothesis has failed but a new "statistical TOL hypothesis" is promising. The TOL heuristic usefully organizes data without positing fundamental evolutionary truth.

**Reviewers:**

This article was reviewed by W. Ford Doolittle, Nicholas Galtier and Christophe Malaterre.

## 

"And after a while you'll hear a deep voice saying, "Neighbor, how stands the Union?" Then you better answer the Union stands as she stood, rock-bottomed and copper sheathed, one and indivisible, or he's liable to rear right out of the ground."

*The Devil and Daniel Webster *(Stephen Vincent Benet, 1937)

"On a huge hill,

Cragged, and steep, Truth stands, and he that will

Reach her, about must and about must go"

*Satire III *(John Donne, written 1593-1600)

## Looking back to go beyond the Tree of Life

One might think that there is nothing further that could be said about the last decades of debate on the Tree of Life (TOL). There is certainly no need to recapitulate the numerous overviews of these debates as they set out the positions of key participants (e.g., [1-3]). However, as the pageantry of 2009's "Darwin Year" subsides, it seems to be an appropriate time to reflect on where TOL studies are headed in relation to the legacies on which they draw. We will consider two key issues: the specific effects of these debates on the conceptual frameworks of TOL studies, and how these frameworks are actually used. We will discuss whether they function as hypotheses or heuristics. To put it in a deliberately over-simplified way, it seems useful to examine two basic questions:

• What are reasonable interpretations of the TOL in the postgenomic era?

• What is the utility of the TOL for research in evolutionary biology and perhaps beyond?

## The legacy

The natural starting point for thinking about the TOL is Darwin's explication of its metaphorical power in *On the Origin of Species*.

"The affinities of all the beings of the same class have sometimes been represented by a great tree. I believe this simile largely speaks the truth. The green and budding twigs may represent existing species; and those produced during each former year may represent the long succession of extinct species.

... As buds give rise by growth to fresh buds, and these, if vigorous, branch out and overtop on all sides many a feebler branch, so by generation I believe it has been with the great Tree of Life, which fills with its dead and broken branches the crust of the earth, and covers the surface with its ever branching and beautiful ramifications" [4].

In subsequent revisions of his central ideas, Darwin did not make more concrete how to conceptualize or construct the TOL. Despite the appeal of other aspects of Darwinian thinking for many biologists of the era, the TOL did not immediately stimulate many efforts to represent the totality of organismal history in the form of evolutionarily connected branches. One notable exception was Ernst Haeckel's depiction of a universal TOL on the basis of a three-domain genealogy of Protista (including Monera), Animalia and Plantae [5]. However, the stage-like evolutionary relationships inherent in Haeckel's phylogeny, coupled with older religious connotations of TOL imagery, appear to have inhibited the scientific uptake of his universal tree [6-8]. Even subsequently, the classification of organisms continued primarily as a taxonomical rather than an evolutionary activity, with branching patterns of specific lineages simply being derived from existing taxonomical schemata [9-11,8]. This situation continued well into the twentieth century despite the advent of the "new systematics" in the 1940 s. "Our phylogenies are invented to account for our taxonomic facts or theories", grumbled botanist Harry Allan [12]. Nevertheless, the TOL as a general unifying representation of the totality of specific phylogenies persisted, even if it was not realized in practice.

The development of contemporary phylogenetic methods in the 1960s and 70s, especially the formalized principles of cladistics, resulted in a revolution in phylogenetic practice. However, for evolutionary microbiology, this transformation mattered very little. It took the combination of these phylogenetic methods with an even more revolutionary source of data -- molecular sequences -- to bring microbes and particularly prokaryotes into the embrace of a Darwinian system of classification. Once universal characters were available for all organisms, the Darwinian vision of a universal representation of all life and its evolutionary history suddenly became a realistic possibility. Increasing reference was made to this universal, molecule-based phylogeny as the "comprehensive" tree of the "entire spectrum of life" [13-17].

However, somewhat paradoxically, the very process of building phylogenies with molecules revealed the extent of horizontal gene transfer (HGT), and thereby threatened the TOL concept in regard to its core ideas of a unique ever-bifurcating branching pattern. Inter- and intra-species reticulation was a problem even when limited genetic datasets were available, but became a major issue with the advent of genomics in the 1990s. While genomic data has massively enabled comparative evolutionary analyses of microbes [18]), it has simultaneously exposed the mosaic nature of archaeal and bacterial genomes and the sheer amount of HGT that has occurred over the course of evolution (e.g., [19-22]. Although this reticulation is most extensive in the evolution of prokaryotes, eukaryotes have also increasingly been caught in the act [23-26]. The comparative infrequency of HGT in the eukaryote part of the biological world means, however, that in this case the conceptual implications for the TOL might not be as drastic: the evolutionary histories of many eukaryotes appear to produce tree-like patterns (e.g., [27]). However, by definition, the TOL is supposed to be the tree of *all *life and *all *evolution, so it is conceptually and epistemically misleading to discount non-tree-like evolution when such processes occur in the majority of life-forms and history of life

In response to the predicament of the prokaryote part of the TOL, researchers have positioned themselves in conceptual camps in regard to how they deal with the challenge of HGT [1,3]. While according greater or lesser importance to HGT is one way to approach prokaryote evolution, a more constructive stance may be conceivable now that methods and concepts have developed even further. In what follows, we attempt to identify the conceptual frameworks and epistemological strategies deployed in evolutionary studies as responses to the deluge of data produced by genomics and metagenomics, and to show how these strategies have produced revised understandings of the TOL. Throughout this article, we strive to distinguish clearly between the ongoing discussions of the ontological status of the TOL (concerned with what it is) and the epistemological strategies in TOL research (concerned with how TOL knowledge is produced and how it can be used in other research). These two perspectives on the TOL have often been conflated but we attempt to show why it is worthwhile to separate them.

## Conceptual frameworks: Reinterpreting the TOL

**Figure 1:**
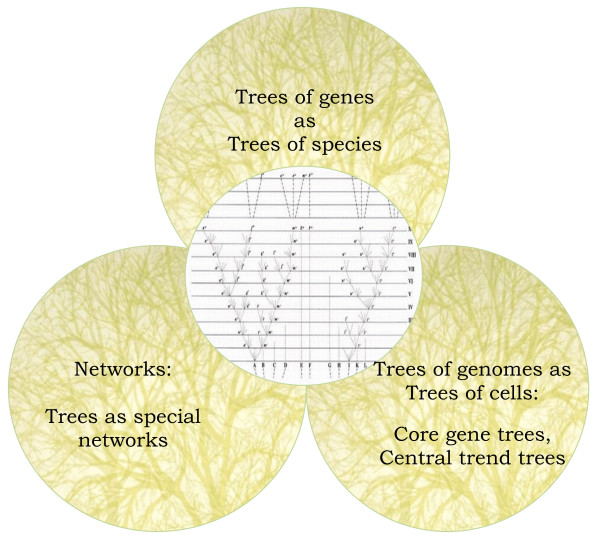
**Conceptual frameworks of the TOL in relation to Darwin's tree simile**.

Many substantial advances have been made in phylogenetic methods, including the development of sophisticated evolutionary models, tree-building techniques (including faster tools suited to the analysis of genome-wide data sets) and reliability estimates of tree inferences, as well as databases and other computational tools. In this section, we are primarily concerned with the concepts that underpin these methods and their respective results. Specifically, our focus is how the TOL has been reconceptualized in light of the fact that the more molecular data is analysed, the more difficult it is to interpret straightforwardly the evolutionary histories of those molecules. Rather than renouncing the universal tree, many evolutionary biologists have instead elected to restructure their understanding of the TOL in relation to bodies of data and what can be done with them. We will outline a variety of positions that encompass an ever more extensive range of modifications to the basic TOL concept (Figure 1). These stances range from "business as usual" on the basis of finding clear signals of the one true TOL, to a perspective in which local trees are seen as just occasional structures in the "real" web of life. All these positions draw on Darwin's tree metaphor, and they also overlap and feed into one another in various ways, but each commands a distinct conceptual space for itself.

### 1. Trees of genes as trees of species

Trees of gene and protein sequences are typically considered most valuable when they can be justified as representing trees of species. To achieve this representational status, a gene or a set of genes have to meet some criteria of genealogical markers. The first two related criteria are the most obvious ones: i) a gene has to be (nearly) universal, i.e., represented by readily recognizable orthologs (preferably single-copy) in all cellular life forms; ii) the sequence of the gene in question has to be sufficiently conserved to allow the construction of an unambiguous alignment and an informative tree. The third criterion is more controversial and harder to apply: a gene used for the construction of a reference tree has to be minimally prone to HGT. Genes favoured under these criteria include those for ribosomal RNA, ribosomal proteins, elongation factors, RNA polymerases and several other (nearly) universal, highly conserved genes [28,29]. A few of these markers are considered to be so evolutionarily "special" that they have become the basis of reference trees for the whole TOL [30,15]. The problems of the most well known reference trees, the trees of 16S and 18S rRNA genes, have been frequently discussed (e.g., [31,32]). Nevertheless, for many evolutionary biologists, the concept of a reference tree can still be justified as long as its limitations are understood (e.g., [33]).

However, researchers -- even if they continue to use reference trees -- increasingly recognize that single-gene trees, and even composite multi-gene trees, might obscure more than they reveal. These trees cannot take into account non-bifurcating patterns from major evolutionary events, such as endosymbiosis, co-evolving symbioses, hybridization and any other occurrences of lineage fusion [34-37]. More generally, HGT is now recognized as a major factor of evolution in the prokaryote world. Treating all these non-tree-like processes as problems that obscure the "true" TOL greatly skews and limits the understanding of evolutionary history that is one of the central goals of evolutionary biology -- along with understanding processes and patterns of evolution [38].

The second way of relating gene trees to species trees is to think of the gene trees as contained "within" the species tree. This route is particularly attractive for the systematics of organisms for which there is already a widely accepted phylogenetic placement in the TOL (primarily multicellular eukaryotes), but it has also had appeal for prokaryote phylogenetics. An obvious problem is that the species tree has to be "predetermined" in order to pick and construct the right gene trees (e.g., [39,40]), and this makes the phylogeny circularly presuppose its conclusion. However, as with the previous conceptual relationship between gene and species trees, discordance between trees for individual genes -- not just in prokaryotes, and not just because of HGT -- has also led to fundamental questions about whether gene trees could be simply understood as tracing a history "within" a known species tree [41-43]. "In considering these issues", wrote phylogeneticist Wayne Maddison,

"one is provoked to consider precisely *what is phylogeny*. Perhaps it is misleading to view some gene trees as agreeing and other gene trees as disagreeing with the species tree; rather, all of the gene trees are part of the species tree, which can be visualized like a fuzzy statistical distribution, *a cloud of gene histories*" [41].

Rather than gene trees being contained within species trees or standing in for them, new concepts of the TOL and of evolutionary history in general began to be articulated with the increasing availability of comparative genomic data. Because the species tree is generally perceived as the true aim of phylogeny (or at least used to be until recently), new modelling techniques have been devised and broader treatments of data developed in order to represent the species tree less problematically. Given the abundance of molecular data, a major investment has been made in attempts to reconstruct trees of genomes. In the process, the very concept of "species tree" (and thus TOL) has been revised.

### 2. Trees of genomes as trees of cells

Under the broad banner of phylogenomics, efforts to reconcile inconsistent data and resolve the branching order of all lineages of life have been developed and championed [44]. Phylogeneticists are obliged to believe that traces of vertical signal can be detected amongst the evolutionary noise (although these very categories imply certain expectations) and so are torn between interpreting such signal as the central truth of the evolutionary history or as an indication of limited genetic relatedness that is not necessarily central to our understanding of evolution. A major outcome of the attempts to understand the relationship between putative signal and noise in genomic data has been the generation of new concepts of the TOL. While there are several methodological routes involved [45,46]), two streams of genome tree construction illustrate this tension due to their substantially different underlying ways of thinking about the TOL.

Core genome approaches are concerned with an evolutionarily stable core of genes that can be taken to represent the organismal lineage, which is seen as the process of binary genome replication and cell division (thus justifying the tenet of bifurcation). In accordance with the previously mentioned criteria for the choice of reference genes, this approach seeks to identify genes that are widely represented in genomes, and most importantly, that produce congruent phylogenetic signals (e.g., [47-52]). A degree of success has been achieved under this conceptual framework (different methods may be employed), with the identification of universal genes that appear to track the same evolutionary story. There are many questions, however, about whether the trees generated for this purpose, particularly concatenated sequence trees, are methodological artefacts [53], and whether such analyses say much about the TOL or simply produce a partially distorted history of several genes.

Perhaps the biggest problem with such an approach is how well the identified cores represent the evolutionary history of the organisms and genomes that contain them. The (nearly) universal gene core of cellular life is extremely small and functionally skewed. One much scrutinized core analysis examined genomes of 191 species from all three domains of life but was able to identify only 31 universal genes, primarily, those for ribosomal proteins [54]. Prokaryote genomes typically contain between 1,000 and 4000 genes, so any tree built on the basis of 31 genes is a highly reduced representation of the intended TOL -- "a tree of 1%" in a famously trenchant criticism [36]. More generally, the fact that all genes in prokaryote genomes are likely to have experienced at least one HGT event in the 3.5 billion year history of cellular genomes means that no pristinely untransferred core exists [55]. The core approach might, therefore, be better interpreted as concerned with a "least transferred" subset of genes. In that case, the core would be a "fuzzy" gene set displaying a particular statistical trend, rather than a precisely defined set, and this is the conceptual space another version of the genome-based TOL inhabits.

Central trend approaches are built on the quantification of more and less transfer. They combine individual trees of genes in order to foreground vertical tree patterns against the much more complicated backdrop of the "forest" of life [56-60]. Such conceptualizations factor in the pervasiveness of HGT but search for an indicative message of vertical descent from the composite data. This trend, composed of the most universal signal, can usually be picked up only faintly at deep phylogenetic levels, except for the signal of bifurcation between archaea and bacteria [57]. It may not be possible, ultimately, to recover any other details of deep branching and even tree tips may remain in doubt for some lineages [55,61,62]. Nevertheless, for some of these supertree constructions, a "modal information" TOL seems to emerge strongly enough to be a "backbone" tree that is merely draped with some fine "cobwebs" of HGT [60].

While none of these analyses see the central trend as the majority signal in the forest, they do recognize it as an extremely important one. In one case, when using a specially designed "tree-net trend" score, the central tree-like trend amounts to approximately 40% of the total information on prokaryote evolution [58]. But is such a "statistical tree" what is traditionally meant by the TOL? This was certainly not how the TOL was conceived in the first era of molecular phylogeny before the recognition that different genes might have distinct evolutionary histories. The statistical TOL approach also involves the acknowledgement that averaging out the signal from different gene trees may produce artefactual trees while obscuring relevant aspects of evolution [63]. The willingness to make this transition may have more to do with the perceived epistemological function of the TOL (which we explore below), than with commitments to the ontology of the tree (e.g., its "realness").

## Trees of cells in light of trees of genomes

Challenges to the TOL have caused many builders of genome trees to conceive differently of their reconstructive aims. Rather than seeing the TOL exclusively as a tree of *species*, based on trees of genes, the TOL has been appealed to as a tree of *organisms *or a tree of *cells *(e.g., [49,57,64-67]). The justification for refocusing TOL efforts in this direction is that the history of life is, as Darwin knew, a history of bifurcating cell divisions as well as one of genome replication (which he didn't know). If the TOL topology is taken to represent the history of cell divisions, then it can conveniently be conceived as the organismal backdrop against which the web-like intricacies of genome evolution can be examined (e.g., [49,51]). For core genome proponents, the tree of cells concept means that incongruence between individual gene trees is diminished in evolutionary importance even if it continues to pose methodological problems. The aim of phylogeny can still be seen as accessing the "true" history of organisms without being distracted by the aberrant history of some parts of the genome [51]. Some core advocates go so far as to proclaim that the cell-based conception of the species tree would be valuable even if it were incongruent with every single constructed gene tree (e.g., [68]). They do assume, however, that many gene trees will, in fact, be reconciled with the "supposedly known" species tree [52,68]). This use of the tree-of-cells concept revives the original problem of conceptualizing gene trees as representing or contained by species trees. It requires the assumption and circular reasoning that the species tree (now disguised as a tree of cells) is already available whereas one of the major purposes of constructing and comparing gene trees (for prokaryotes at least) is to infer the species tree.

From the central trend perspective, the tree of cells is not concretely envisaged as true history that in some way operates as a reference tree. What the tree of cells conception is doing for this group of phylogeneticists is mediating the construction of genome trees and species trees by making another stepping stone of inference. The argument is based on a claim that tree thinking is inherent in biology because the replication of genetic material is itself inherently tree-like [69]. Under this view, even when the incongruence between numerous gene trees is taken into account, genes can still be considered as fundamental units of evolution. Accordingly, the goal of phylogenetics -- in this context perhaps more appropriately denoted phylogenomics -- is perceived as deciphering any signs of distinct structure that might exist in the "phylogenetic forest of life" (i.e., the compendium of all gene trees). Despite the complications of finding appropriate methods, some of these trends are taken to represent vertical descent, a concept on which this approach and phylogenetics more generally continue to rely [57,58].

### 3. Webs of genomes and trees of genes

A growing response to the difficulties in prokaryote phylogeny is to embrace the view that there is no universal tree that subordinates all other evolutionary patterns. Trees for individual genes or possibly sets of genes may exist, from this viewpoint, but only as partial representations of much more complex evolutionary processes, especially in the prokaryotic world [70,71]. The most radical position is that hierarchical patterns and apparent phylogenetic congruence may result from non-tree-like processes (i.e., systematically biased HGT), and that at the very least, this alternative needs to be explored [72-75]. The last several years have seen considerable effort put into the development of network models and representations of their outputs (e.g., [76,71,63]). Representation is a major problem for such approaches, with the complexity of many networks requiring that certain data or processes are filtered out just for the network to make interpretable sense [77].

In the face of all this effort in reconceptualizing the evolutionary process, the problem persists of whether making too many theoretical concessions to the existence and widespread nature of HGT undermines the very aim of phylogenetics and systematics, and thereby prematurely forecloses on a better understanding of the relationships between microbes, the TOL, and evolution in general. We think this question is central to the debates and developments of the last decade, and the theme of this collection of papers in *Biology Direct*, "Beyond the Tree of Life", could just as well be framed as a question. But what makes the debate persist and continue to offer important points of discussion may be due as much to how the TOL is conceived to function in the research it inspires as to how it can be elucidated in the context of evolving biological entities.

## The role of evolutionary trees and the TOL in creating knowledge

The TOL has obviously undergone a number of conceptual transformations as it has encountered new data and methods. As a core or central trend, for example, the TOL is quite far from early molecular TOL concepts, in which vertical descent by gradual modification was the supposition governing analysis of the data. But it is not just the representational status of the TOL that has changed -- from trees of genes as trees of species, to trees of genomes as trees of cells, to trees as special networks -- but its very epistemological status. Phylogeny in general has made major shifts over the last decades. Throughout the 80s and 90s, and into the 2000s, the understanding of what molecules could do for phylogeny moved from a role as tools that could test existing theories about evolutionary relationships to becoming the substance of evolutionary analysis and the source of novel hypotheses about the evolutionary history of organisms [78].

From one point of view, the extent of these epistemological and conceptual transformations suggests that evolutionary biologists or at least investigators of microbial evolution should see the TOL as a ladder to be kicked away. As a conceptual prop and an epistemological tool, the TOL has taken its users as far as it could, and further habitual use will be merely misleading, say Ford Doolittle and Eric Bapteste [70]. According to this view, network-based approaches should replace the TOL in order to understand the evolutionary history of groups of organisms [79]. The force of this argument depends, however, on how the TOL is being used and whether any of those uses can be justified.

As we noted in earlier, a distinction should be made between the epistemology of the TOL and its ontology, or nature of its existence in the world. Quite frequently, the two are conflated: the postulated existence of the TOL means that it must be straightforwardly justified as knowledge. Richard Dawkins illustrates this conflation with his usual panache:

"For there is, after all, one true tree of life, the unique pattern of evolutionary branchings that actually happened. It exists. It is in principle knowable. We don't know it all yet. By 2050 we should -- or if we do not, we shall have been defeated only at the terminal twigs, by the sheer number of species" [80].

But, at least as far as prokaryote evolution goes, it is obvious that representing it in the form of the "one true tree of life" will be very difficult, if not impossible, and that parts of the TOL may never be justifiably extracted from the range of evolutionary signals that can be detected [71,81]. Even when a tree-like central trend is detected in the "forest of life", it is recognized that deep internal branches tend to be extremely short (the so-called compressed cladogenesis [CC] model). As noted by Pugibò et al., "a consistent phylogenetic signal seems to be discernible throughout the evolution of archaea and bacteria but, under the CC model, the prospect of unequivocally resolving the relationships between the major archaeal and bacterial clades is bleak" [57]. Moreover, given the pivotal role of endosymbiosis accompanied by massive gene transfer from the endosymbiont to the host, as well as sporadic HGT, representation of the entire history of eukaryotes as a single tree is not possible either [82].

What Dawkins does articulate effectively in his statement above are the background assumptions to the idea of a universal TOL, with the implications that knowing it is crucial to understand evolution and biodiversity. This deeply held intuitive conviction holds, as Darwin did, that all evolution involves groups arising out of groups, and that every organism should belong to (at least) one of those groups. While all evolutionists would accept such claims, they would not agree on how much of this process can be known, and whether it is the primary representation that should subordinate all other evolutionary processes.

Doubts about the extent to which the TOL can be known are conjoined with questions about how the TOL actually functions in evolutionary biology. There are several candidates for the epistemological role the TOL plays in evolutionary biology, and these include the functions of an axiom, a hypothesis or theory, a model, or a heuristic. We will examine two of these possibilities with the particular aim of understanding why the TOL endures despite the evidence against it, and whether one of these epistemological accounts of the TOL enables us to understand better the conceptual transitions and debates that have transformed it over the last 150 years.

### a. The TOL as hypothesis

The TOL, although usually described in general and unifying terms, may also rendered as a hypothesis. Ford Doolittle and Eric Bapteste describe the TOL as a hypothesis and go on to point to its apparent weaknesses in regard to standard expectations about hypotheses [70,79,83]. Although they also mention that the TOL is a "model", and a "heuristic epistemological model", their main characterization includes qualities routinely associated with hypotheses, namely testability and falsifiability [70]. They see the TOL as a hypothesis composed of three connected propositions: natural selection (and potentially other processes) drives modification, descent occurs with modification, and hierarchical patterns are produced by that process. Because of its multi-propositional nature, it also appears sensible to view this account of the TOL as a model (the distinction between models and hypotheses is not important for our discussion here, but can be useful in other contexts, e.g., [84]) or a theory. However, because broader theoretical statements need to be dealt with as specifically testable claims, we will accept the designation of hypothesis for the way in which phylogeneticists have tended to treat TOL claims.

Doolittle and Bapteste's criticism of the TOL is not on the grounds that it is a hypothesis, but that it has not been sufficiently treated as one. They believe that the refutation of the TOL hypothesis has not been accorded enough attention, and that starting with Darwin, the historical wielder of the TOL, researchers have used the data to be explained (the apparent hierarchy of the relationships between organisms) as evidence of the explanation (the hierarchical branching structure of life as a consequence of descent with modification).

"The body of data (the explanandum) for which a hypothesis (the explanans) proposes to account cannot at the same time constitute proof for that hypothesis, nor can further data of the same kind" [70].

Put even more emphatically, TOL logic can be phrased as "natural classifications are tree-like *because *the process that produces the characters on which they are based are tree-like" (see Review 1 below). According to Doolittle and Bapteste, this circularity means that proper testing has been abandoned, and that the difficulties with the TOL in modern phylogenetics can be attributed to the fact that it has not been duly treated as a hypothesis. If it had been, Doolittle and Bapteste contend, then the TOL hypothesis would indeed have been rejected, and brand new hypotheses formulated and tested.

This idea of treating tree branches as conjectural hypotheses is something that cladists enthusiastically introduced into phylogeny when Hennig's systematics was translated linguistically and conceptually into a broader sphere of phylogenetic systematics. Cladists have tended to rely heavily on philosopher's Karl Popper's ideas of falsification (and corroboration), which has led to many oversimplifications of scientific practice [85,86]). One major problem in this equation of hypothesis with falsifiable conjecture is that it is usually not the case that a hypothesis is abandoned or substituted when it is "falsified". Much more commonly, the original hypothesis is modified to accommodate otherwise conflicting findings [87,88]). This is what seems to have happened to the TOL hypothesis, as findings of HGT have stretched original expectations of the TOL [1,3]. Indeed, under the "forest of life" approach, the path to accommodation of the TOL as hypothesis seems straightforward. We can postulate the existence of gene trees, which is indeed a natural assumption given the intrinsic bifurcating character of the replication process (see above). Then, we can formulate the falsifiable "statistical TOL" (STOL) hypothesis: is there a single, significant central trend in the "forest of life", i.e., a single-tree topology that is, on average, most similar to other topologies in the forest? A rigorous test of the uniqueness of the STOL topology is technically demanding and still to be performed but preliminary analyses strongly suggest that such a trend exists [57]. Thus, the accommodating STOL hypothesis seems to meet this particular falsification criterion. Does this approach get rid of the circularity emphasized by Doolittle and Bapteste? The answer depends on whether the hypothetical STOL and the trees for individual genes are treated as data of the same kind or of different kinds. The latter view seems reasonable because, as pointed out above, a gene tree is a natural implication of the bifurcating replication process but the existence of a STOL does not follow from this assumption in the face of HGT.

Doolittle and Bapteste, if pressed on this point, could claim that accommodation and expansion are not legitimate ways in which to treat hypotheses, especially when doing so relies on circularity between explanans and explanandum. Circularity has been a frequent problem for evolutionary biology, and has been a charge made against the supposed claim that natural selection can be measured by survival of the fittest [89,90] as well as against aspects of phylogenetic classification [91]. Philosophers have often discussed Darwin's reasoning as "inference to the best explanation", according to which explanation comes before inference, and observations thus legitimately support the hypothesis meant to explain them [92]. But worries about circularity (even if avoided by the STOL) might be irrelevant because the TOL could have another epistemological role, that of a heuristic, for which the route of inquiry is rather more complex than hypothesis testing or even inference to the best explanation.

### b. The TOL as heuristic or a tool

For many purposes, the TOL serves as a general metaphor of evolutionary relatedness, despite the fact those relationships cannot be captured by an exclusively bifurcating pattern. Even if not strictly justified, the TOL construed in this way has the capacity to depict and organize distinctions between cell types, genome structure, fine-grained genetic architecture, physiology, habitat and many other features that are central to the evolution of life on earth. From a practical point of view, the TOL provides a framework in which to order biological knowledge for both scientific and broader social purposes. It is a tool with which to explore a range of phenomena, some of which it identifies and the rest of which it may indicate cannot be captured by that particular approach. More specifically, the TOL, all its limitations notwithstanding, is necessary as a scaffold for reconstructing scenarios about the evolution of features of organisms (such as various functional systems). Arguably, this is key goal of evolutionary research, and it is unclear how it can be achieved without using a tree-like framework. This sort of use could be thought of as heuristic.

William Whewell, a natural historian who had considerable influence on Darwin's view of science, is generally credited with one of the earliest elaborations in English of the term "heuristic". Whewell thought that if he could not 'treat the Art of Discovery as a kind of Logic, [he] must take a new name for it, Heuristic' [93]. He proposed that heuristics provide scientists with a "bond of unity by which the phenomena are held together" with the consequence that the topic may be examined more closely and formally. Perhaps the most influential contemporary account of heuristics can be found in the work of Hungarian mathematician, George Pólya. Pólya advocated heuristic reasoning in his classic 1945 book, *How to Solve It *[94]. He described heuristics as "serving to discover", and outlined how such reasoning is "provisional and plausible only" with the aim of discovering the solution to particular problems. Heuristics, on the basis of induction or analogy, produce a partial solution that may become more complete. This way of thinking has been taken up by various scientific communities, including computer scientists (e.g., [95]), cognitive scientists and Artificial Intelligence researchers [96].

One of the most useful background sources for understanding the TOL as a heuristic comes from Darwin himself, as he investigated this concept. When he began to use the metaphor ("simile") of the TOL, he used it in a heuristic sense, as a tool or an aid to evolutionary classification:

'As it is difficult to show the blood-relationship between the numerous kindred of any ancient and noble family, even by the aid of a genealogical tree, and almost impossible to do this without this aid, we can understand the extraordinary difficulty which naturalists have experienced in describing, without the aid of a diagram, the various affinities which they perceive between the many living and extinct members of the same great natural class' [4] (emphases added).

In this passage, in the final full chapter (Chapter 14) of *The Origin*, Darwin is expanding on the nature of classification in regard to his theory of natural selection. He is talking about the pragmatic value of understanding descent with modification with the help of genealogical trees. Moreover, Darwin follows Whewell's description of hypotheses as

'"of service to science, [even] when they involve a certain portion of incompleteness, and even of error. The object of such inventions is to bind together facts which without them are loose and detached ... even if they themselves somewhat misstate the matter."

[97-99]). But this is not what is understood by hypothesis today, and not what Doolittle and Bapteste meant when they talked about Darwin's TOL hypothesis. In today's phylogeny, heuristics are discussed almost exclusively in computational terms and hypotheses are more rigidly defined as propositions that are right or wrong. Cladism's addiction to bifurcating logic and falsifiability [100], on top of the much earlier shift in biology from natural history to experimentation, is probably the reason that evolutionary heuristics such as the TOL have been converted into hypotheses to be tested [101]. It is important to keep in mind that cladist "hypotheses" are not couched at the TOL level, however, and that it took the advent of molecular phylogeny to bring back the idea of a unifying and unified phylogeny. But as Woese and other phylogeneticists revived Darwin's grand pattern of a great tree, they treated it not only as a general representation of the total evolutionary history of life, but also as a tool with which to probe heuristically further molecular data. Thus, the TOL functioned in the original Darwinian sense as an aid in that it connected and made general sense of otherwise localized evolutionary relationships.

Some of the revealing residues of this way of thinking heuristically about the TOL can be found in contemporary statements that expose the TOL function as far from that of a strictly "falsifiable" hypothesis. For example:

"The only way to discover whether HGT could destroy Darwin's dream of understanding the great kingdoms of nature is to assume that it cannot, and then make every effort to try to determine the tree of life" [102].

"Whether or not this central trend is denoted a tree of life could be a matter of convention and convenience, but the nature of this trend as well as the other trends that can be discerned in the forest merit investigation" [57].

In other words, statements such as these leave open the ontological status of the tree (the nature of its existence) in favour of treating it as an epistemological tool that might generate and justify new knowledge. Not only has the study of the TOL given further reason to pursue such investigation, but also TOL limitations are now understood more clearly. Even some strong TOL advocates are able to agree that any construction of a universal tree is in fact a heuristic that is useful for exploring evolutionary patterns and processes.

"In my view, a tree is just a human-made conceptual tool that we might decide to adopt if it *means *something to us, like any other graphical representation, irrespective of its 'existence' in the real world" [103].

This is a conventionalist viewpoint, or one that accepts the TOL as a pragmatic tool and not a natural category. Thinking of the TOL as a heuristic allows a conceptual rapprochement to be forged between TOL researchers who uphold strong claims about the universal tree, and network researchers who are opposed to imposing the TOL globally to represent prokaryote evolution. As one of the latter explains,

"I have no objection to the continued use of an rRNA tree (or of any other agreed upon averaging or gene core-based TOCD&S [tree of cell division and speciation]) as a conventional framework for *classification*, provided everyone knows that that is all that it might be, a conventional taxonomic framework, not the TOL with all its baggage. Other ways of classifying microbes (for instance by gene content or ecological role or indeed by relative position in a multidimensional network) might well have more predictive value, but still this relatively stable hierarchical scheme would serve a very useful organizing function. In fact, I think this is the posture that many microbiologists have already accepted" [104].

Conceiving of the TOL's epistemological function as heuristic fits this pragmatic stance very well. It is a function that is flexible enough to appeal broadly and could explain why the TOL is thriving as a research topic (captured by PubMed abstracts, for example [105]) notwithstanding the many challenges to its realness and epistemic legitimacy. Rather than being put off by these problems, evolutionary biologists are increasingly drawn to efforts to understand broader swathes of evolutionary relationships. The persistent appeal of the TOL could also have something to do with the increased attention to Darwin in 2009 (the 200th anniversary of his birth, and the 150th of the publication of the *Origin*), but more important in the long run is the power of the TOL to probe copious data, suggest high-level explanations, and make general sense of the information produced by an explosion of tools, analyses and models in evolutionary biology. Heuristics need not explain or capture everything: their epistemic importance lies in their ability to open up valuable lines of inquiry.

It is useful to recall that heuristics can be deployed in a variety of ways. In some cases, the TOL heuristic might function as a positive probe in which it connects together local phylogenies; in others it might be used as a negative heuristic, when researchers use the TOL to seek phenomena to which it cannot apply (much of the HGT research, for example). And as we have shown already, heuristics such as the TOL are not frozen in epistemic time. As new evolutionary findings and interpretations have arisen from initial heuristic-driven research, the TOL has accommodated new ways of probing evolutionary patterns and processes. Today's TOL explores molecular and other levels of evolution in the company of network heuristics. Does this mean the TOL is too plastic and accommodating a heuristic? Only in the same way that the human mind is able to deal with positive and negative findings by adjusting models and techniques on the basis of preceding success and failure. In this respect, heuristics embody major features of scientific practice and general cognitive activity.

It is possible that for the more ardent TOL critics, presenting it as a heuristic may seem like a desperate strategy that is part and parcel of old and doomed "tree thinking" [106]. And for many other TOL researchers, the TOL is something *more *than a heuristic -- it is a true model or theory that is isomorphic to evolutionary reality. Thinking of the TOL as a heuristic will not solve any problems of its application when the TOL is discussed in these idealized forms but it may help understand why idealizations are not the only way to proceed.

### c. Pluralism versus monism

From a more radical point of view, holding the TOL as hypothesis or heuristic is already part of the problem because it is deemed to have no singular epistemic status, and no privilege as a representation of evolutionary history. This perspective holds that contemporary understandings of HGT mean that it is time, finally, to give up on any "unifying metanarrative" such as the tree [70], or that it may be appropriate to find an alternative, such as a Web of Life [79,71] or a Ring of Life [37].

Sometimes couched in terms of the conflict between pluralist and monist perspectives, the TOL debate is seen by these proponents as moribund [70]. To replace tree-thinking, they argue for a more accommodating framework in which trees are located in a much more tangled forest, and the emphasis is placed on achieving a genuine understanding of the forest. Trees are part of the picture but far less than the main part. This pluralistic perspective also suggests an additional issue of whether to separate representations and theories of eukaryote and prokaryote evolution, due to the different tempos, modes and outcomes of evolution involved [70,71]. More than one account of evolutionary processes and mechanisms may be necessary to encompass the varieties of evolving life on the Earth.

The trouble with this perspective for many researchers is that it may be giving up too much because there is no well-organized or obvious alternative to the TOL, and piecemeal descriptions of aspects of evolution are conceptually dissatisfying for many biologists. It is possible that this gap will be filled by "Web of Life" models, tools and concepts. However, there may still be some life left in the TOL in regard to how it poses a particular set of scientific questions and brings together a diverse community of scientists seeking closer understanding of evolutionary processes and relationships. We think that the variety of ways in which the TOL has developed, both conceptually and epistemologically, indicate its continued usefulness. Although ongoing loyalty to the TOL is unlikely to be founded primarily on essential core markers of tree-like evolution, its future could instead involve much fuzzier conceptualizations and more flexible epistemological strategies. It is not at odds with pluralism for researchers to continue to investigate heuristically aspects of this fuzzy TOL. On the contrary, it would be anti-pluralist to insist that such investigations should not proceed.

## The future standing of the tree

What does it mean then to talk about going "beyond" the TOL? The general question that might be raised at the end of such an overview is whether the TOL is still relevant. Does it continue to stimulate programmes of research with novel findings and methodological innovations? And is further debate about the TOL constructive? As we have noted, some of those involved in the discussions believe it may be time to lay the debate to rest.

"All sides express confidence in their positions, and the debate often seems to be at an impasse" [70]

We think that our analysis shows the very opposite of an impasse. Although in some respects, idealized positions continue to be articulated, in the main TOL research -- whether skeptical or convinced of the TOL's existence, constructability and relevance -- is about shifting conceptual frameworks and flexible accounts of what the TOL is and does. A common basic understanding of evolution has been profoundly enriched by insights into the dynamic evolution of genomes, in which a wide range of processes and entities including diverse mobile elements and specific mechanisms for HGT play major roles (e.g., [107-113]). Wrestling with the conceptual and epistemological function of the TOL has played an important role in engendering these new ideas.

From a practical perspective, TOL research is an excellent illustration of a successful research programme [87]. As a heuristic, it has transformed previous understandings, and been conceptually modified along the way. The TOL not only guides further investigation but also manages to organize numerous disciplines and research perspectives. Does "going beyond" the TOL mean abandoning such progress? We think that reflecting on the TOL's conceptual and epistemological status is part of the process by which a research programme continues to reinvent and transform itself in light of earlier insights. Such reflections also clarify some of the debate around the TOL. If it is seen as a hypothesis -- something that can be precisely rejected and then abandoned forever -- it can be the cause of some impatience that this hasn't occurred when the basic TOL hypothesis has been refuted. But if the TOL is understood and used as a heuristic (and there seem to be many contemporary instances of this sort of use), then the value of its continued deployment is such that no mere "falsification" will get rid of it. Rather, if it continues to advance understanding of evolution, even when found wanting or in need of qualification, then it would be wasteful to discard it. Some of the debate may indeed be symptomatic of the very different epistemological expectations of the TOL, rather than the opposition of different conceptual frameworks.

We hope that our overview shows not only the wealth of conceptual and epistemic value produced by the TOL, but also that it illustrates how good science works. The universal tree is not and has never been purely about right and wrong facts, but about the pragmatic knowledge-producing capacity of the TOL framework. The current state of debate indicates that the TOL still has conceptual and epistemic worth. It may be fought over, but never announced as a dogma free of requirements for evidence and demonstration. The TOL is very much alive in this sense, and we predict its demise is far from imminent. However, the future of the TOL is not today's TOL. Transformations in regard to concepts and epistemological function are underway already, and this thematic series on "Beyond the Tree of Life" will indicate some of them.

## Conclusions

The irrefutable demonstration by phylogenomics that different genes in general have distinct evolutionary histories made obsolete the belief that a phylogenetic tree of a single universal gene such as rRNA or of several universal genes could represent the "true" TOL. However, this irrevocable realization does not immediately dispose of the TOL, which can be reconceptualized in at least two distinct ways. First, the TOL can be treated as an evolutionary hypothesis. The refutation of this hypothesis in the original, strong form, as a single faithful representation of the evolution of organisms, has prompted its modification to the "statistical TOL hypothesis". The existence of a statistically significant tree-like trend in the "forest" of individual gene trees is a testable proposition that still has to be investigated in detail. Second, the TOL can be deployed as a heuristic for evolutionary studies in which a tree of just a single universal gene can be extremely useful as long as one realizes that it is only a convenient framework for organizing data rather than a fundamental truth about evolution. These two modified accounts of the TOL are only partially independent: corroboration of the statistical TOL will certainly reinforce its use as a heuristic, and further heuristic application will lead to modification of the statistical TOL conceptions. Looking "beyond the TOL", we are inclined to believe that the use of the TOL as a heuristic to organize and analyse comparative data and partial trees will be with us for the long haul. Whether there is life remaining in the TOL beyond this usage or whether it has to be replaced by new, probably web-like representations of genome evolution, is a question of major interest for phylogenomic studies and will continue to inspire research far into the future.

## Competing interests

The authors declare that they have no competing interests.

## Authors' contributions

Both authors contributed equally to the planning and writing of this Review. Both authors read and approved the final manuscript.

## Reviewer Comments

### Reviewer 1

W. Ford Doolittle (Dalhousie University, Halifax, Canada)

Although there is much about this paper with which I wholeheartedly agree, and which I think admirably summarizes the state of play, the authors do seem a bit confused about what Eric Bapteste and I called Darwin's Tree of Life Hypothesis. In their understanding, we saw "the TOL as a hypothesis composed of three connected propositions: natural selection (and potentially other processes) drives modification, descent occurs with modification, and hierarchical patterns are produced by that process." What we wrote, though was:

"1. The pattern of groups subordinate to groups embraced by a unique inclusively hierarchical classification based on homologies (true affinities in Darwin's language) is indeed not arbitrary. It reflects an underlying natural reality with a natural cause, rather than ''some unknown plan of creation, or the enunciation of general propositions'' in Aristotelian logic, embedded in the practices of systematists.

2. That natural cause is historical, and in particular, it is direct descent with modification, a branching process whose branches will be recaptured in the most truly natural and correct classification, which might in principle be extended to include the last common ancestor (or ancestors) of all extant forms.

3. Modification is driven by natural selection." [70]

I now see this formulation as unduly wordy and prone to the sort of emphasis-altering paraphrasing O'Malley and Koonin offer. Bapteste and I could/should have offered a short form of our own, something like this: natural classifications are tree-like *because *the process that produces the characters on which they are based is tree-like (and selection is the driving force). The emphasis would be on "because", because Darwin's was, I believe, as in these lines from earlier in the same paragraph that O'Malley and Koonin quote:

"The limbs, divided into great branches, and these into lesser and lesser branches, were themselves once, when the tree was young, budding twigs, and this connection of the former and present buds by ramifying branches may well represent the classification of all extinct and living species in groups subordinate to groups."

Jerry Coyne, in his recent *Why Evolution is True *[114], similarly sees Darwin endorsing the Tree as an explanation, or more precisely as a prediction of his theory.

"Actually, the nested arrangement of life was recognized long before Darwin. Starting with the Swedish botanist Carl Linnaeus in [1735], biologists began classifying animals and plants, discovering that they consistently fell into what was called a ''natural'' classification. Strikingly, different biologists came up with nearly identical groupings. This means that these groupings are not subjective artifacts of a human need to classify but tell us something real and fundamental about nature. But nobody knew what that something was until Darwin came along and showed the nested arrangement of life is precisely what evolution predicts. Creatures with recent common ancestors share many traits, while those whose common ancestors lay in the distant past are more dissimilar. The natural classification is itself strong evidence of evolution."

But surely the flaw or "circularity", the conflation of explanandum and explanans here should be obvious. We have a fact (Tree-like classification) and we come up with a theory (Tree-like evolution) to explain the fact. Then what do we predict in order to test our theory? Tree-like classification! Panchen's book, *Classification, Evolution and the Nature of Biology *[115], which caused me first to twig to the circularity issue, does outline some of the ways out of this. And of course *Evolution is True *[114], for those reasons and because of the overwhelming consilience of many lines of evidence.

**Authors' response**: *Our question here would be whether Darwin and subsequent evolutionary biologists are using the logical structure of "X because Y" (explanatory, in that Y explains X). We are not convinced this is the logical structure Darwin was intending and believe it may better be described as abductive reasoning, which was outlined by Charles Peirce in 1903 in the form:*

"The surprising fact, C, is observed;

*But if A were true, C would be a matter of course*,

*Hence [because of C], there is reason to suspect that A is true*" [116].

If we substitute C with hierarchical order and A with TOL, then the abductive structure of TOL reasoning is:

"The surprising fact, of hierarchical order, is observed;

*But if the TOL were true, hierarchical order would be a matter of course*,

*Hence [because of hierarchical order], there is reason to suspect that the TOL is true"*.

*This structure does not involve the "because" reasoning of causal explanation. Rather, it is ampliative in that it adds to what is known and is potentially able to improve understanding of the phenomena of interest. Abductive reasoning has been much discussed in philosophy of science, and has its critics as well as its advocates. More recently, abductive reasoning has been discussed in the form of "inference to the best explanation" as philosopher Peter Lipton *[92]*points out:*

"Inference to the Best Explanation can be seen as an extension of the idea of 'self-evidencing' explanations, where the phenomenon that is explained in turn provides an essential part of the reason for believing the explanation is correct. ... hypotheses are supported by the very observations they are supposed to explain. Moreover, on this model, the observations support the hypothesis precisely because it would explain them."

*In other words, explanation comes prior to inference, and involves an evaluation of how well different hypotheses would explain existing evidence. The problem then becomes one of how to evaluate the "best" explanation, and the logical objection of circularity drops out of the picture (i.e., it's not a matter of one "proving" the other). However, we agree that in the following section, Doolittle is objecting to the outcome of the inference by arguing that the TOL is **not **the best explanation and certainly not the **only **explanation available to account for the "surprising fact" of hierarchical order*.

**Doolittle responds in a second review**: I have only a folk-philosophical understanding to help me deal with this, but I swear that this abductive reasoning formulation has a "because" hidden underneath it. Why else would "hierarchical order" be a "matter of course" if the "TOL were true"? If by 'TOL' the authors mean to refer to the TOL *hypothesis *(as articulated above in bold), then the reason hierarchical order follows as a matter of course is because and only because a tree-like process is expected to produce the tree like-pattern of similarities and differences between organisms that is enshrined in a hierarchical classification.

Seems pretty because-ish to me: there is a causal connection between evolutionary branching and the disposition of traits on which hierarchical classification is based. We would not say, for instance, that "if E = mc^2 ^were true, hierarchical order would be a matter of course" because even though E does equal mc^2 ^we can think of no similar causal connection to hierarchy.

Possibly though, O'Malley and Koonin are not referring to the TOL *hypothesis *when they say "if the TOL were true, hierarchical order would be a matter of course" but to the TOL *itself*, that tree-like pattern of relationships between taxa that molecular phylogeneticists hope to reconstruct (some believing that they already have). Now *that's *circular: it's like saying "If there were a natural (aka true) tree-like or hierarchical pattern of relationships, hierarchical order would be a matter of course".

But the authors' thoughts do persuade me that there must be degrees of circularity. The goodness of fit between hierarchical classification and the TOL hypothesis does seem like some sort of *support*, or *consistency with*, or even *evidence *for it (in the sense that hierarchical classification is none of these for E = mc^2^). But surely it is not "proof". Surely it a standard part of scientific practice to expect that a hypothesis should make predictions about data not yet obtained, and of a different sort than the data which suggested the theory. Francisco Ayala, one of Darwinism's most stalwart supporters, accepts this too, writing that,

"If a hypothesis is formulated to account for some known phenomena, these phenomena may provide credibility to the hypothesis, but by themselves do not amount to a genuine empirical test of it for the purpose of validating it" [117].

I am also persuaded that "Inference to Best Explanation" is a valid way to do science, but also encouraged by what I take is the authors' admission that the existence of other defensible explanations for the same "surprising fact" strengthens our (mine and Eric Bapteste's) claim that whatever Darwin meant by the Tree "simile", it could/should now be taken as an hypothesis. But surely they would not have us believe that when only one explanation is apparent to the scientific community, it must be taken as fact.

Which gets us back to the "surprising fact of hierarchical order." Why "surprising"? Who was surprised (in 1859)? What is the null hypothesis here? Why should we expect disorder rather than order? Darwin's goal was to come up with a naturalistic explanation for an orderly pattern of relationships in nature to replace the supernaturalistic one then (and sadly still) hegemonic. ("Let the earth bring forth grass, plants yielding seed of each kind, and trees bearing fruit of each kind ..."). That there is such an orderly pattern doesn't discriminate between these views, for all we scientists embrace the former.

**Doolittle's first response continued**: But natural classification is not proof of evolution or of the existence of an underlying tree-like process. Not only is the logic shaky, there *is *an alternative. It is in principle possible, as Peter Gogarten and colleagues will argue elsewhere in this issue [118], that biased LGT can produce tree-like patterns from a process that is not itself tree-like. Maybe taxon A and taxon B appear to be sisters to the exclusion of C because they share genes more frequently, not an ancestor more recent. This too would be "descent with modification". So it is instead specifically (1) that descent with modification is a predominantly tree-like process and (2) that this tree-like process produces a tree-like pattern, that together make up Darwin's hypothesis. "Descent with modification" is often taken to mean tree-like evolution, but it needn't, and we probably should not even have used the term in the long-form of the Tree of Life Hypothesis. The word "genealogical" is equally squirrely. When we use it in a human context, unique family trees (usually only patrimonial ones) come instantly to mind, but in fact we are each at the end of 2^N ^genealogies, going back N generations.

I think that when cladists say that trees branches are hypotheses they mean that they are hypotheses about relationships between specific taxa, not a general explanation for why there should be trees at all. So Darwin's Tree of Life Hypothesis, as Bapteste and I understand it, is *not *gently morphable into some looser Statistical Tree of Life (STOL) Hypothesis, as O'Malley and Koonin seem to suggest. An STOL could also arise from rampant gene transfer -- with no deep vertical signal -- provided there is some bias affecting LGT. And who could sensibly imagine that there would not be many biases? But such an STOL would be precisely *not *what Darwin's Tree of Life Hypothesis was about. So O'Malley and Koonin are partly right when they claim that Bapteste and I would "claim that accommodation and expansion are not legitimate ways in which to treat hypotheses." They would be fully right had they added "especially when the expanded hypothesis has transmogrified into something antithetical to its original form."

**Authors' response**: *We tend to agree regarding the drastic and irreversible transformation of Darwin's TOL hypothesis and had already noted in the text the specific nature of cladist hypotheses. Darwin simply was not equipped to think of anything other than an organismal/species tree. He could not think of trees for individual genes because he was unaware of the existence of the latter. We note that Doolittle agrees with us regarding what we said about accommodation being rejected by Doolittle and Bapteste, but think the even stronger clause tacked on in this response would be hard to defend. It is not clear that the extended TOL hypothesis should be considered "antithetical" to Darwin's ideas even though it is indeed dramatically different. From today's perspective, Darwin's hypothesis is not properly defined as such a definition was outside the purview of the science of Darwin's day. With this realization, the purported circularity of the argument becomes less evident and less damning (even if abductive reasoning is not taken into account) -- we think it actually goes away. The fundamental tree-like character of evolution is not derived from an observed tree-like pattern of classification. On the contrary, this is an intrinsic feature of the evolutionary process that follows a fundamental observation of biology, namely the bifurcating character of gene replication *[69]*and cell division. Under this view, the STOL can be conceived as a straightforwardly testable hypothesis. If anyone manages to show that there is no statistically significant trend of topologically congruent trees, the STOL will be found wanting in terms of predictive power and effectively refuted. So far it seems to have withstood the test. We agree that the biased HGT hypothesis of Gogarten, Doolittle and Lawrence is a viable alternative although there seems to be considerable uncertainty as to whether biased HGT could be the *sole *explanation for the observed tree-like patterns for gene ensembles, or just a complementary explanation to a genuine trend of vertical evolution *[32,119]. *Testing this hypothesis poses conceptual and technical challenges but some approaches have been attempted, and the results seemed to contradict the most radical version of the biased HGT hypothesis *[58]. *However, most of the work in this direction remains to be done. This is the main point we would like to make in this discussion: the STOL might turn out to be false but it is a bona fide hypothesis*.

**Doolittle responds in a second review**: Well, OK, maybe "antithetical" is too strong, and of course it's a mug's game guessing what Darwin would think were he still here. And I will agree that the STOL hypothesis as stated above (that there are more congruent trees than expected by chance), though possibly unprovable at depth, is not only coherent but unavoidably true. But if -- as is possible -- the majority of the genes that make up any prokaryote's genome, determine its phenome and define its "relationships" to other prokaryotes actually arrived in that genome by lateral transfer rather than vertical descent, then I'd put money on the venerable bicentenarian saying "Oh, that's not what I had in mind at all!"

**Doolittle's first response continued**: It's not easy to argue against the value of the TOL as an heuristic or tool, but I'll try. If what undergirds such a TOL (which would be in actuality an STOL) are central tendencies, then we must always be careful to hedge statements we make about it. If we say something like "halophilic archaea arose from within the methanogens with the importation of bacterial genes for respiration" we need to be very careful to note that methanogens that far back in time need not have had any specific genes or capacities other than a taxon-defining 16S rRNA. To draw what I think is an apt analogy, we need to be similarly careful when we say something like "early English arose from several ancient Germanic languages" not to pretend that we would recognize it or any of those others if someone started speaking them to us on the street. The literature of language evolution is amazingly parallel to our own on these questions, but ahead of us in sophistication and realism, I think. For instance, Heggarty et al. in a 2011 paper on Germanic languages [120] say this about the evolution of English:

"If our real goal is to uncover the histories of the *populations *that spoke given languages, rather than abstract schemas intellectually satisfying for their binary purity, then it is served by using language data to arrive at a picture of the nature and degree of cohesion (or otherwise) of speech communities within a language family, through the story of its divergence. To this end, we must represent the historical and linguistic truth that English ultimately underwent a longer and more total isolation than did most continental varieties from each other. The approach that best uncovers this signal is to measure and represent the full impact of *all *those far-reaching changes that came to mark it out as so distinct from all other Germanic varieties in so many ways ... Furthermore, while English is widely assumed to have derived from a *mixture *of Germanic dialects--eminently logical also in terms of population history--this too cannot be represented in a tree model. That structure is inherently forced to oversimplify the most plausible history. Nor could it capture the clear possibility that of the dialectal mix that went to make up English, a greater part was of more western than northern Germanic character and provenience, but not *exclusively *so, especially at a time when the difference between those two groups may well have been very much a continuum still. It is such matters of degree, rather than mutually exclusive binary alternatives, that speak in favour of distance and network analyses for language history, and against tree-only ones."

We microbiologists must accept that this sort of messy historicity will also be appropriate for any true description of microbial evolution, however tightly we hug our statistical trees.

**Authors' response**: *We too accept the inescapable messy historicity of any adequate description of evolution, especially (but by no means exclusively) when it concerns microbes. We are perhaps slightly at odds about whether statistical analyses can capture in a helpful way the fuzziness of evolutionary processes. Our reasoning is this: given such messiness, if we want to discover any patterns in this history, an organizing framework is badly needed, and the (S)TOL is one of the best frameworks for such a purpose. The STOL can be used as a framework for the construction of evolutionary scenarios, which is what much of evolutionary biology is about. The ultimate value of this framework is contingent on the robustness of the STOL hypothesis (see above). If the hypothesis stands up to rigorous examination and testing, the STOL can be reasonably considered a uniquely powerful heuristic. Should the hypothesis fail to stand up against such scrutiny, a tree for a universal gene like 16S RNA still can be used as a standard of comparison, without bestowing on it any special ontological status. Certainly, our advocacy of the STOL as a framework for evolutionary studies should not be taken as an attempt to invalidate promising *complementary *frameworks such as various kinds of evolutionary networks *[121,122].

**Doolittle responds in a second review**: It would be silly to claim that trees are not useful in the construction of evolutionary scenarios. But to the extent that any STOL is not the history of the lineages of organisms or genomes such studies address, but rather a summation of biases in LGT, we can be misled.

### Reviewer 2

Nicolas Galtier (CNRS, Montpellier, France)

I much enjoyed reading this piece, which I think is a sound, balanced, well-written overview + opinion about an important topic -- Tree of Life (TOL) significance. The manuscript correctly makes clear that

(i) genomic evolution is far from 100% tree-like,

(ii) drawing a tree does not mean assuming that genomic evolution is 100% tree-like,

and reviews the way these two basic-looking statements have been acknowledged, and their relative importance weighted, by the scientific community. I feel I agree with most of the arguments made in the paper, some of which I tried to express in a previous *Biology Direct *review [103]. I share the prediction that TOL will remain useful to evolutionary sciences in the forthcoming years, or decades. Here are a couple of thoughts that arose while reading this article, which I take the liberty to express although not all of them are tightly connected to the specific content of the manuscript.

The text says: "understanding of evolutionary history arguably should be the goal of all research in evolutionary biology". I would modulate. I think a large part of research in evolutionary biology is about the process, not the pattern. Besides history, we also would like to understand the evolutionary forces having shaped current biodiversity, including mutation, selection, drift, recombination, duplication, HGT, speciation, extinction, vertical inheritance. I note that the last five of these processes (i) implicitly acknowledge the relevance of the tree of cells, (ii) would be optimally studied if the tree of cells was known, and (iii) have been typically approached by taking the "average"/"central" tree as an estimate of the tree of cells - sometimes in a disguised way, by calling it "taxonomy".

**Authors response**: *We fully agree and have modified the text in question to state:*

*"Treating all these non-tree-like processes as problems that obscure the "true" TOL greatly skews and limits the understanding of evolutionary history that is one of the central goals of evolutionary biology -- along with understanding processes and patterns of evolution *[123].*"*

I would like to suggest that "falsifying" the TOL in the face of HGT would be equivalent to, e.g., falsifying the Linnean taxonomy in the face of gene flow, genetic incompatibilities, and hybridization. We know that the living world is not made of reproductively isolated groups of fully interbreeding individuals. Yet the species concept captures a lot of the information about genetical exchanges between living things (at least in animals and plants). We can debate the concept, refine it, call it insufficient, but we just cannot dismiss it.

**Authors' response**: *Agreed. The use of the term "falsification", with its connotations of an exact hypothesis that can be decisively falsified by a single or even a few negative findings is problematic. We had noted that in the text, but had used the word casually in a few places ourselves. We have replaced these uses of falsification with broader terminology except where we cite others who use this exact term. And we think our discussion of heuristics harmonizes with the reviewer's thoughts on usefulness and information capture*.

I wonder why the discovery of HGT in prokaryotes led to such extreme proposals as "Darwin was wrong" and "the TOL is dead", whereas the discovery of gene and genome duplication, gene loss, transposition, and incomplete lineage sorting, and other efficient anti-TOL processes, did not. If you think about it, the widely accepted vertebrate tree is a "tree of a few percent". The proportion of nucleotides in the human genome for which the personal history during the last 500 million years matches the canonical vertebrate tree is minute. Nobody complains when, in vertebrates, people discard paralogues (which they circularly identify thanks to prior speculations about the species tree), repeated elements and genes of viral origin, then build trees. In this taxon, it is undisputed that the species tree is a meaningful representation of evolution, however incomplete it may be, and however prevalent and interesting the anti-TOL processes may be (and they really are).

So why this difference? I think one reason is that in vertebrates we have a mental representation of evolving units (species) independently of their genomes. The species tree makes sense when you are interested in species. We would much like to learn about our vertebrate ancestors, irrespective of the fraction of genes we have vertically inherited from them. Vertebrate species are so neatly defined that we are happy to ignore the genealogical mosaicism of their genome, at best considered as a curiosity. Bacterial species, in contrast, are merely seen as identical bags of non-identical genes. Consequently, what matters in the first place (to us) in bacteria is the forest of gene trees, not the tree of bags. This might indeed reflect a genuine biological difference between the two groups (e.g., a more complex genotype-phenotype link in vertebrate than in bacteria), such that the TOL would actually have less scientific value in bacteria than in vertebrates. Alternatively, the difference of treatment might reflect our much deeper knowledge of (or interest in, or ability to perceive) the vertebrate than the bacterial biology and diversity.

**Authors' response: ***We find this to be a deep comment a full discussion of which would require another manuscript perhaps the same size as this review article. We can offer only a few brief thoughts here. It is true that the gulf between the current perceptions of the status and importance of trees for vertebrates (and even animals in general), on one hand, and for bacteria and archaea, on the other hand, is huge. We can think of at least four reasons (certainly, not mutually exclusive) why this is the case. 1. To be fair, the vertebrate tree only becomes a "tree of a few percent" if the entire genome is considered. Looking at protein-coding genes only (indeed, less than 1.5% of mammalian DNA), the pan-vertebrate gene set is respectable (thousands of genes), and if in-paralogues are carefully taken into account, something like a "tree of half the genes" is possible, with topologies for individual genes being highly congruent. All the importance of the non-tree-like evolutionary processes notwithstanding, this is a far cry from the "tree of one percent" that is feasible for prokaryotes. 2. The vertebrate tree is, in a sense, "tangible", with internal nodes often associated with specific forms about which much is known from the fossil record. There is no fossil support for the tree of prokaryotes, and no chance ever to obtain such support, which certainly makes this tree less "real" than the tree of vertebrates. 3. For vertebrates, the concept of species is clearly defined and biologically meaningful despite some limitations. For prokaryotes, the species concept is fuzzy at best and meaningless at worst, its applicability appears to vary for different groups of bacteria and archaea, and there is no chance of a universal, biologically sound definition of species *[124]. *Thus, if one chooses to speak of a "species tree", it is clear what is meant in the case of vertebrates but not in the case of prokaryotes. 4. The difference in the perception of trees is psychological and indeed reflects common and serious under-appreciation of the non-tree like aspects of evolution that are present and important in all life forms*.

This leads me to my very last comment. The text says:

"This pluralistic perspective also suggests an additional issue of whether to separate representations and theories of eukaryote and prokaryote evolution, due to the different tempos, modes and outcomes of evolution involved. More than one account of evolutionary processes and mechanisms may be necessary to encompass the varieties of evolving life on the Earth."

I fully agree with the second sentence especially. HGT is one out of several anti-TOL evolutionary processes, and eukaryotes, by the way, are champions of HGT (partly, but not uniquely, through endosymbiosis) and of genome mosaicism. I am confident we would not question the relevance of the vertebrate tree even if we discovered substantial HGT in this group. I suggest that the distinct values we assign to the species tree in distinct taxa is determined by our representation of what species are, not by the way species evolve. If the genome of an insect and a *Wolbachia *strain once became physically merged and fully co-transmitted, we would comfortably call the resulting organism an insect. If such a fusion occurred between a bacterium and an archaeon, we would probably call the resulting organism a new domain of life. Non-tree-like evolution is everywhere. Scientists decide how meaningful it is.

**Authors' response: ***Another truly interesting point. It is difficult to argue with the example given by the reviewer. Certainly, in these two cases, the interpretations of genome fusion would be quite different. Are the decision criteria "purely" subjective? Perhaps, they do not have to be. One criterion that immediately comes to mind is quantitative: if one of the merging genomes (the one designated as donor) makes a contribution that is orders of magnitude less than the contribution of the other partner (recipient), we will confidently identify the recipient as the main organism in the resulting chimera. In contrast, if the contributions of the partners are comparable, we will speak of a new domain or some such. This approach certainly works in the cases of Wolbachia-insect and bacterium-archaeon. However, it is easy to come up with counterexamples: about half of mammalian genomes consist of remnants of retrotranscribing elements but we have no problem at all classifying these organisms as vertebrates. Similarly, certain mesophilic archaea such as Methanosarcina acquired more than 20% of their genes from bacteria but all microbiologists agree that they remain archaea. Thus, the quantitative criterion is not entirely satisfactory, and a qualitative one may prove more appropriate. When genome fusion leaves the cell organization of the host (more or less) intact, we are comfortable to interpret the situation as "horizontal gene transfer as usual" -- these are the cases of Wolbachia and insect, Methanosarcina, and retroelements in vertebrates. By contrast, when genome fusion is accompanied by (or perhaps causes) a drastic change in cell biology, we are inclined to speak of the emergence of new domains or at least of major new groups of organisms. These are the cases of endosymbiosis in eukaryotes that lead to the emergence of new organelles, such as mitochondria and plastids. This qualitative criterion seems to make biological sense and applies also to the interpretation of trees. When a tree for a set of "core" genes reflects the continuity of cellular (organismal) biology, that tree remains relevant, even as one has to realize that it describes the evolution of a small fraction of the genetic material in the respective organisms. In contrast, when the biological continuity breaks down, so does the tree. In some senses, this distinction implies that ontological priority is given to cellular organization over genetic structure, and that morphological considerations trump molecular ones. There are potentially good epistemological reasons for accepting such hierarchies, but as pointed out by Galtier, it is of deep interest to reflect on how historical and psychological factors may have a role to play in preferences for certain explanations*.

### Reviewer 3

Christophe Malaterre (Institut d'Histoire et de Philosophie des Sciences et Techniques, CNRS, France)

In this paper, O'Malley and Koonin very convincingly argue that the notion of "Tree of Life" (TOL) covers two distinct concepts: (A) a hypothesis about the representation of the evolution and relatedness of organisms on Earth, (B) a heuristic tool that is useful to classify these organisms and that leads to new knowledge in evolutionary biology. In the first section of the paper, the authors review and clarify two main ways of understanding what the TOL is: the TOL notion may cover trees built from molecular phylogenetic studies of single genes, in particular the 16S and 18S rRNA genes; it may cover also trees built from the congruent phylogenetic signals of several genes, be they part of a "core genome" or chosen for the "central trend" that they exhibit. The authors then briefly compare these TOL tree-meanings to the "web of genomes" (or network) approach. In the second major section, they develop their main thesis on the two different concepts covered by the notion of TOL. First, the TOL can be understood as a scientific hypothesis. In this case, under a strict interpretation, this hypothesis has been refuted; yet under a modified interpretation that accommodates new findings into a "statistical TOL", this hypothesis still awaits refutation and is even useful. Second, the authors argue that the TOL can be understood as a heuristic tool, that is to say a concept that is not to be evaluated as true/false with respect to its correspondence to nature, but that is useful to the practice of science in at least two ways: as a conventional framework for classifying organisms or species, and as a means to "open up valuable lines of inquiry". In this case too, the TOL notion proves useful. In a short third section, the authors develop the idea that "the universal tree is not and has never been purely about right and wrong facts, but about the pragmatic knowledge-producing capacity of the TOL framework".

I find the conceptual clarifications of the TOL proposed by the authors extremely useful and enlightening. It is indeed crucial to realize that the TOL can cover at least two radically distinct concepts -- scientific hypothesis and heuristic tool -- and that it may therefore be evaluated in at least two different ways: with respect to its refutability or with respect to its usefulness. As such the paper is an extremely valuable conceptual contribution to the more general TOL debate.

Let me mention however two points that raise questions. The first one has to do with the notion of "hypothesis". Some may indeed argue that the TOL is not just any type of "hypothesis", but a hypothesis that tells us something about the real evolutionary relatedness among groups of organisms. In other words, it might be argued that the TOL is above all a scientific *theory*. Of course, following Poincaré [125], one may say that all generalizations and theories are hypotheses; yet they are *particular *types of hypotheses: hypotheses that are refutable while also well corroborated by observations [126], that include bridge principles that mediate their correspondence to observation data [127], that take the form of research programs constituted by a central core of propositions surrounded by a belt of protective assumptions [87], or even hypotheses that are constituted by classes of models isomorphic to certain domains of nature [128]. It seems to me that taking a "theory" perspective on the TOL -- a perspective that is often implicit in the paper since it is indeed made reference to Popper and Lakatos among others -- could help the argument proposed by the authors in at least two respects. First, some philosophical models of scientific theories make it possible to account for theory modification or replacement over time; such a view could thereby help explain why the TOL is still around despite claims of its "refutation". Second, philosophical models of scientific theories also make it possible to discuss how theories relate to the real world (for instance via bridge principles); and a discussion of how the TOL as a hypothesis/theory does indeed relate to nature could indeed extend the argument of the paper, even if the authors purposefully restrain their arguments to non-ontological matters.

**Authors' response**: *These are very useful thoughts on what it is that is being disputed in regard to the TOL. We have discussed above with our first reviewer (WFD) whether the TOL took the logical form in Darwin's thinking that Doolittle and Bapteste suggest it did. In this article, we mentioned the multi-propositional nature of Darwin's ideas about the TOL. These connected propositions, both core and contextual, might be most accurately described as a theory, as Malaterre suggests, and we could then engage in extended philosophical discussions of the correspondence of the TOL theory to aspects of the world. The TOL debates are both about the realness of the TOL and about its epistemological value, and in this article we argue that the two facets of the debate can be cleanly and profitably separated. Theories are something philosophy of science has focused on for decades. This attention has been illuminating but it has also obscured many other aspects of scientific practice. One of these has been how theories or core elements of theories are operationalized in workaday hypotheses such as claims about the TOL. This sort of activity is at least part of what we address in this article. Indeed, it would be useful to analyse the TOL as a theory, and to see how different philosophical accounts of theory interpret TOL claims and contestations, but pragmatic concerns guide our choices here. The extended discussion that Malaterre advocates, in which the TOL is examined as a theory, would no doubt be illuminating for TOL researchers*.

A second point concerns the fact that, as shown by the careful and well-crafted section on "reinterpreting the TOL", the notion of TOL may be explicated in many different ways. As a result, some may argue that their ways of defining a TOL differ from those that are offered in the paper and that, as the result, they may not map onto the two roles identified by the authors (hypothesis or heuristic tool). It seems to me that a specific risk stems from a possible conflation of the epistemology of the TOL and its ontology. This point is clearly identified by the authors, yet only in the second section of the paper, and I wish this distinction were made earlier. Indeed, when presenting the different construals of the TOL in the first main section, the authors mention "trees of genes as trees of species" and "trees of genomes as trees of cells". Yet these expressions tend to conflate different possible meanings associated with the TOL: Is the TOL a tree of genes or a tree of species? What does the "as" imply? Is it rather a tree of genomes or a tree of cells? If it is a tree of cells, how does it differ from a tree of species?

**Authors' response**: *This comment points to a dimension of discussion we largely sidestepped and yet which is of enormous importance to TOL debates. We now mention earlier in the manuscript how we think ontological issues have been conflated with epistemological ones, and we also announce there that we try to separate the two as best we can. The questions Malaterre asks in this part of the review lie at the heart of ontological clarification of the TOL, and as far as we are concerned, they are unresolved. What we are doing with the "as", therefore, is signalling that we are agnostic on the exact nature of the relationships between the two interpretations of the TOL. We think it unlikely that there is an identity relationship (in which "as" would mean "the same as"). We note that methods are being used that presume some sort of equivalence, and we then focus on those methodological choices. Laura Franklin-Hall *[67]*has done some philosophical work on the implications of such relationships, but there is still more that could be done, as Malaterre points out. This is especially because in practice, TOL ontology and epistemology are not isolated from one another. And yes, it is likely that our two foci, hypothesis and heuristic, do not exhaust what can be done with the TOL. Indeed, Malaterre rightly emphasizes the fruitfulness of thinking about the TOL as theory, and the same could no doubt be said for the TOL as a model*.

Maybe distinctions could be made between different types of TOL depending on the types of entities that they refer to. One might, for instance, distinguish three types of entities: (i) *real source entities *such as genes, core genomes, gene sets that are extracted from particular present-day organisms and whose data are used to generate a tree-like or network-like representational model; (ii) *represented entities *that would include the source entities (as end-points in the model) but also such entities as ancestral genes or gene sets (as additional nodes in the model); and (iii) *interpreted entities *such as organisms, species, past or present, once an interpretation of the model is given. Accordingly, the TOL might be understood as a *represented entities TOL *(a tree-like model that is built from the source entities using various computational tools, some of which might in addition include hierarchical branching assumptions), or as an *interpreted entities TOL *(an interpretation of the previous tree-like model in terms of organisms or species for instance). Furthermore, one may consider the existence of the real genealogy of the real organisms on Earth, and therefore postulate the existence of *real entities phylogenies *at different levels (genes, genomes, cells, organisms, species). It then seems that some of the controversies mentioned in the paper -- in particular when it comes to the falsifiability of the "TOL as hypothesis" -- could be understood as disagreements about how to answer three different questions: (1) Given a set of *real source entities*, is the inferred *represented entities TOL *true -- in the sense that if the corresponding *real entities phylogeny *were known, the two would match -- or is it false due, for instance, to lack of data or to wrong inferences made by our computational tools? (2) Given a *represented entities TOL *(for instance a gene tree), is the *interpreted entities TOL *(for instance a species tree) true -- in the sense that if the corresponding *real entities phylogeny *were known, the two would match -- or is it false due to a wrong interpretation? And (3), are the *real entities phylogenies *tree-like or not? And, while the third question is a question that concerns the structure or topology of phylogenies, the two first questions concern the more general problem of inference justification. These three types of questions might also help clarify the different epistemological levels at which the TOL might play a role as a hypothesis/theory, and more specific circumstances in which it might endorse a more heuristic role.

**Authors' response**: *This is an interesting and potentially useful tripartite scheme for further analysis of debates about the TOL, and we believe such a framework of inquiry has never been suggested before. While it is not practical to rewrite the present article with this structure in mind, we would heartily endorse any attempt (from philosophical or life-science standpoints) to analyse TOL claims and research in light of this intriguing framework*.

In any case, the conceptual distinctions that O'Malley and Koonin propose are most relevant to the debate: distinguishing between the TOL as a hypothesis (or a theory) and the TOL as a heuristic will help make discussions on the topic more precise and will improve the conceptual framing of some well-entrenched controversies.

**Authors' response**: *We are most grateful to all three reviewers for the exceedingly thoughtful analyses they made of our paper, and the new routes of inquiry they have shared with us*.
